# Estimated Drug Overdose Deaths Averted by North America's First Medically-Supervised Safer Injection Facility

**DOI:** 10.1371/journal.pone.0003351

**Published:** 2008-10-07

**Authors:** M-J. S. Milloy, Thomas Kerr, Mark Tyndall, Julio Montaner, Evan Wood

**Affiliations:** 1 British Columbia Centre for Excellence in HIV/AIDS, St. Paul's Hospital, Vancouver, Canada; 2 School of Population and Public Health, University of British Columbia, Vancouver, Canada; 3 Department of Medicine, University of British Columbia, Vancouver, Canada; Norwegian Knowledge Centre for the Health Services, Norway

## Abstract

**Background:**

Illicit drug overdose remains a leading cause of premature mortality in urban settings worldwide. We sought to estimate the number of deaths potentially averted by the implementation of a medically supervised safer injection facility (SIF) in Vancouver, Canada.

**Methodology/Principal Findings:**

The number of potentially averted deaths was calculated using an estimate of the local ratio of non-fatal to fatal overdoses. Inputs were derived from counts of overdose deaths by the British Columbia Vital Statistics Agency and non-fatal overdose rates from published estimates. Potentially-fatal overdoses were defined as events within the SIF that required the provision of naloxone, a 911 call or an ambulance. Point estimates and 95% Confidence Intervals (95% CI) were calculated using a Monte Carlo simulation. Between March 1, 2004 and July 1, 2008 there were 1004 overdose events in the SIF of which 453 events matched our definition of potentially fatal. In 2004, 2005 and 2006 there were 32, 37 and 38 drug-induced deaths in the SIF's neighbourhood. Owing to the wide range of non-fatal overdose rates reported in the literature (between 5% and 30% per year) we performed sensitivity analyses using non-fatal overdose rates of 50, 200 and 300 per 1,000 person years. Using these model inputs, the number of averted deaths were, respectively: 50.9 (95% CI: 23.6–78.1); 12.6 (95% CI: 9.6–15.7); 8.4 (95% CI: 6.5–10.4) during the study period, equal to 1.9 to 11.7 averted deaths per annum.

**Conclusions/Significance:**

Based on a conservative estimate of the local ratio of non-fatal to fatal overdoses, the potentially fatal overdoses in the SIF during the study period could have resulted in between 8 and 51 deaths had they occurred outside the facility, or from 6% to 37% of the total overdose mortality burden in the neighborhood during the study period. These data should inform the ongoing debates over the future of the pilot project.

## Introduction

Illicit drug overdose remains a leading cause of death and disability in many urban settings worldwide [1]–[3]. For injection drug users (IDU), the annual rate of fatal overdose is estimated to between one and three per cent per year and is the primary contributor to mortality rates many times higher than in non-IDU populations [4]–[7]. Thus, interventions to reduce mortality from overdose are central to efforts to reduce the harms of illicit drug use.

In Vancouver, Canada, the number of fatal overdoses reached unprecedented levels at the end of the 1990s, especially in the Downtown Eastside (DTES) neighbourhood, the site of an open drug market and explosive outbreaks of HIV and hepatitis (HCV) [8]. In response, North America's first medically supervised safer injecting facility (SIF), named Insite, opened in the DTES in March, 2003. The pilot facility has been the subject of a comprehensive evaluation [9], [10]. Among its findings are a significant increase in uptake to drug and alcohol treatment [11]; a decrease in local measures of drug-related disorder, including public injecting [12]; and a reduction in the prevalence of risk factors for HIV infection, such as syringe sharing [13].

In addition to these public health objectives, the facility aims to reduce the risk of death for clients by providing prompt and appropriate medical attention in the event of an on-site overdose [9]. We have previously reported that the annual incidence of non-fatal overdose in a representative sample of SIF users was approximately 20 per 1,000 person years [14]; and that while on-site non-fatal overdoses were common during the first 18 months of Insite's operation (1.33 per 1,000 injections), none resulted in a fatality [15]. Despite the international evidence suggesting a beneficial effect of SIF on overdose mortality [16]–[18], we are unaware of any peer-reviewed studies that quantify the number of deaths prevented by such a facility. These estimates are obviously important for cost-effectiveness studies and other indicators. Thus, we sought to estimate the number of overdose fatalities averted in Vancouver's SIF.

## Results

From the initiation of the SIF database on March 1, 2004 until February 6, 2008, there were 766,486 injections in the facility, resulting in 1004 overdose events (1.31 per 1,000 injections, or 0.63 per day) in the facility. None resulted in death. The physical manifestations, the substances consumed and the responses taken by staff to these overdose events are shown in Table 1. Of the 1004 overdose events, 453 (45.1% or 0.28 per day) required the provision of naloxone, a 911 call and/or an ambulance, and were included as potential fatal overdoses in our analysis. In approximately 68% of on-site overdose events during the study period, the primary substance injected was heroin, followed by cocaine (17%).

**Table 1:** SIF overdose events by year, substances used, characteristics and interventions

	2004^ = ^	2005	2006	2007	2008^Φ^	ALL
OD events	189	246	230	201	138	1004
ODs/day	0.62	0.68	0.63	0.55	0.76	0.63
Injections	136,971	178,787	178,847	183,989	87,892	766,486
ODs/injection^‡^	1.38	1.38	1.29	1.09	1.57	1.31
**OVERDOSE: SUBSTANCES USED**						
	*n (%)*	*n (%)*	*n (%)*	*n (%)*	*n (%)*	*n (%)*
Cocaine	39 (20.6)	48 (19.5)	44 (19.1)	24 (11.9)	14 (10.1)	169 (16.8)
Crack cocaine	4 (2.1)	1 (0.4)	1 (0.4)	5 (2.5)	2 (1.4)	13 (1.3)
Dilaudid	5 (2.6)	5 (2.0)	4 (1.7)	2 (1.0)	3 (2.2)	19 (1.9)
Heroin	132 (69.8)	164 (66.7)	140 (60.9)	144 (71.6)	103 (74.6)	683 (68.0)
Methadone	2 (1.1)	1 (0.4)	1 (0.4)	4 (2.0)	0 (0.0)	8 (0.8)
Crystal meth	1 (0.5)	0 (0.0)	2 (0.9)	5 (2.5)	1 (0.7)	9 (0.9)
Morphine	3 (1.6)	5 (2.0)	3 (1.3)	8 (4.0)	2 (1.4)	21 (2.1)
Speedball	11 (5.8)	30 (12.2)	22 (9.6)	15 (7.5)	10 (7.2)	88 (8.8)
Talwin & Ritalin	0 (0.0)	1 (0.4)	0 (0.0)	0 (0.0)	0 (0.0)	1 (0.1)
**OVERDOSE: CHARACTERISTICS**						
	*n (%)*	*n (%)*	*n (%)*	*n (%)*	*n (%)*	*n (%)*
Unable to speak	9 (4.8)	44 (17.9)	48 (20.9)	60 (29.9)	51 (37.0)	212 (21.1)
Passed out	8 (4.2)	37 (15.0)	28 (12.2)	27 (13.4)	24 (17.4)	124 (12.4)
Limp	78 (41.3)	120 (48.8)	118 (51.3)	109 (54.2)	78 (56.5)	503 (53.1)
Face blue/pale	71 (37.6)	123 (50.0)	120 (52.2)	119 (59.2)	45 (32.6)	478 (47.6)
Breath slow	106 (56.1)	145 (58.9)	125 (54.3)	117 (58.2)	72 (52.2)	565 (56.3)
Breath stopped	24 (12.7)	52 (21.1)	38 (16.5)	51 (25.4)	23 (16.7)	188 (18.7)
Chest tightness	4 (2.1)	6 (2.4)	3 (1.3)	6 (3.0)	1 (0.7)	20 (2.0)
Seizure	18 (9.5)	15 (6.1)	45 (19.6)	30 (14.9)	8 (5.8)	116 (11.6)
Vomiting	1 (0.5)	1 (0.4)	3 (1.3)	5 (2.5)	2 (1.4)	12 (1.2)
Choking	1 (0.5)	5 (2.0)	3 (1.3)	3 (1.5)	3 (2.2)	15 (1.5)
Sweaty/hot skin	16 (8.5)	31 (12.6)	24 (10.4)	42 (20.9)	28 (20.3)	141 (14.0)
Cold skin	33 (17.5)	37 (15.0)	43 (18.7)	43 (21.4)	28 (20.3)	184 (18.3)
Other	24 (12.7)	12 (4.9)	21 (9.1)	24 (11.9)	13 (9.4)	94 (9.4)
No response to verbal stimulus	22 (11.6)	83 (33.7)	84 (36.5)	71 (35.3)	61 (44.2)	321 (32.0)
No response to pain stimulus	33 (17.5)	110 (44.7)	87 (37.8)	69 (34.3)	58 (42.0)	357 (35.6)
**OVERDOSE: RESPONSES**						
	*n (%)*	*n (%)*	*n (%)*	*n (%)*	*n (%)*	*n (%)*
911 call	54 (28.6)	123 (50.0)	95 (41.3)	93 (46.3)	58 (42.0)	423 (42.1)
CPR	1 (0.5)	4 (1.6)	1 (0.4)	0 (0.0)	0 (0.0)	6 (0.6)
Oxygen	152 (80.4)	202 (82.1)	188 (81.7)	176 (87.6)	92 (66.7)	810 (80.7)
Artificial respiration	18 (9.5)	54 (22.0)	45 (19.6)	45 (22.4)	19 (13.8)	181 (18.0)
Ambulance	14 (7.4)	27 (11.0)	32 (13.9)	33 (16.4)	22 (15.9)	128 (12.7)
Naloxone 0.4 mg	35 (18.5)	89 (36.2)	59 (25.7)	56 (27.9)	46 (33.3)	285 (28.4)
Naloxone 0.4 mg×2	23 (12.2)	44 (17.9)	25 (10.9)	25 (12.4)	24 (17.4)	141 (14.0)
Airway inserted	7 (3.7)	50 (20.3)	50 (21.7)	46 (22.9)	26 (18.8)	179 (17.8)

^= ^March 1, 2004 to December 31, 2004/^Φ^January 1, 2008 to July 1, 2008/^‡^OD events per 1,000 injections

From 2004 to 2006, the British Columbia Vital Statistics Agency reported 32, 37 and 38 annual drug-induced deaths in the Downtown Eastside. The median number of drug-induced deaths per annum from 1998 to 2006 was 38.3. The model inputs for the number of fatal overdoses for each year of the study period, as well as all other model inputs, are reported in Table 2.

**Table 2:** Observations and model parameters for Monte Carlo simulation

	2004^ = ^	2005	2006	2007	2008^Φ^	ALL
**OBSERVATIONS**						
DTES OD deaths	20.1	27.8	28.5	28.7	14.4	137.7
SIF OD events	71	126	99	95	62	453
**MODEL PARAMETERS**						
IDU in DTES	*N* (4700, 500)					
Non-fatal OD rate	Scenario 1: *N* (0.05, 0.01)					
	Scenario 2: *N* (0.2, 0.01)					
	Scenario 3: *N* (0.3, 0.01)					

^= ^March 1, 2004 to December 31, 2004/^Φ^January 1, 2008 to July 1, 2008

Model results, including the DTES fatal overdose rate, the estimated ratio of fatal to non-fatal overdoses in the DTES, the estimated number of non-fatal overdoses in the DTES, and the estimated number of on-site deaths averted, expressed as both a count and a proportion of DTES overdose deaths, are presented in Table 3. The fatal overdose rate in the DTES over the study period was 5.6 per 1,000 person years. Over the same period, the estimated ratio of non-fatal to fatal overdose ranged from 8.9∶1 (given a non-fatal overdose ratio of 50 per 1,000 person years) to 53.8∶1 (given 300 per 1,000 person years).

**Table 3:** DTES fatal overdose rate, non-fatal to fatal overdose ratio and SIF averted deaths from model

	2004^1^	2005	2006	2007	2008^2^	ALL
**DTES fatal OD rate** 3	5.1 (4.0–6.2)	5.9 (4.6–7.2)	6.1 (4.7–7.4)	5.3 (4.1–6.4)	5.3 (4.1–6.4)	5.6 (4.3–6.8)
**NON-FATAL TO FATAL OVERDOSE RATIO**						
Scenario 1^4^	9.7 (5.4–14.0)	8.4 (4.6–12.1)	8.2 (4.5–11.9)	9.4 (5.1–13.7)	9.4 (5.2–13.7)	8.9 (4.9–12.9)
Scenario 2^5^	39.0 (30.1–47.9)	33.8 (26.0–41.6)	32.9 (25.2–40.6)	37.9 (29.1–46.7)	37.9 (29.2–46.6)	35.8 (27.6–44.1)
Scenario 3^6^	58.5 (45.7–71.3)	50.8 (39.6–61.9)	49.4 (38.5–60.3)	56.9 (44.4–69.3)	56.9 (44.4–69.4)	53.8 (42.0–65.6)
**NON-FATAL OVERDOSES IN DTES**						
Scenario 1	195.4 (108.7–282.1)	232.1 (127.8–336.5)	232.9 (127.7–338.2)	232.3 (126.4–338.1)	116.7 (63.9–169.6)	1010.7 (557.5–1463.9)
Scenario 2	784.7 (605.6–963.8)	939.0 (722.6–1155.3)	937.9 (719.1–1156.7)	937.9 (720.5–1155.3)	470.3 (362.1–578.4)	4068.3 (3129.8–5006.8)
Scenario 3	1176.4 (918.7–1434.1)	1408.8 (1099.8–1717.9)	1407 (1097.2–1717.3)	1407.3 (1099.6–1715.1)	705.8 (551.0–860.7)	6108.9 (4770.3–7447.6)
**SIF AVERTED OVERDOSE DEATHS**						
Scenario 1	7.3 (3.4–11.3)	15.1 (1.9–28.3)	12.1 (5.6–18.7)	10.1 (4.3–15.9)	6.6 (3.0–10.2)	50.9 (23.6–78.1)
Scenario 2	1.8 (1.4–2.3)	3.7 (2.8–4.6)	3.0 (2.3–3.7)	2.5 (1.9–3.1)	1.6 (1.2–2.0)	12.6 (9.6–15.7)
Scenario 3	1.2 (0.9–1.5)	2.5 (1.9–3.1)	2 (1.5–2.5)	1.7 (1.3–2.1)	1.1 (0.8–1.3)	8.4 (6.5–10.4)
**SIF AVERTED OVERDOSE DEATHS (PROPORTION OF DTES OD DEATHS)**						
Scenario 1	36.3 (16.9–56.2)	54.3 (6.8–101.8)	42.5 (19.6–65.6)	35.2 (15.0–55.4)	23.0 (10.4–35.5)	37.0 (17.1–56.7)
Scenario 2	9.0 (7.0–11.4)	13.3 (10.1–16.5)	10.5 (8.1–13.0)	8.7 (6.6–10.8)	5.6 (4.2–7.0)	9.2 (7.0–11.4)
Scenario 3	6.0 (4.5–7.5)	9.0 (6.8–11.2)	7.0 (5.3–8.8)	5.9 (4.5–7.3)	3.8 (2.8–4.5)	6.1 (4.7–7.6)
**SIF AVERTED OVERDOSE DEATHS (PER YEAR)**						
Scenario 1	11.7 (5.4–18.0)					
Scenario 2	2.9 (2.2–3.6)					
Scenario 3	1.9 (1.5–2.4)					

1 March 1, 2004 to December 31, 2004

2 January 1, 2008 to February 6, 2008

3 Expressed as deaths per 1,000 person years

4 Scenario 1: Non-fatal overdose incidence is 50 per 1,000 person years or 5% per person per year

5 Scenario 2: Non-fatal overdose incidence is 200 per 1,000 person years or 20% per person per year

6 Scenario 3: Non-fatal overdose incidence is 300 per 1,000 person years or 30% per person per year

Using these inputs, the number of overdose deaths averted in the SIF over the study period was 50.9 (95% CI: 23.6–78.1); 12.6 (9.6–15.7); 8.4 (6.5–10.4) given different estimated rates of non-fatal overdose. The number of averted deaths is equal to between two and 12 per annum over the study period.

## Discussion

Using data from North America's first SIF and published estimates of the rate of non-fatal overdose among active IDU, we derived an estimate of the number of fatal overdoses averted by a supervised injecting facility. Following a Monte Carlo simulation and a three-part sensitivity analysis, the estimates of the number of prevented deaths ranged from eight to 51 from March 1, 2004 to July 1, 2008.

The estimate of the number of deaths prevented is equal to a substantial proportion of the total burden of overdose mortality in the area during the study period. Despite the pilot facility only hosting, by design, approximately five per cent of the daily injections in the DTES, the estimated number of averted deaths was equal to between 6.1 and 37.0 per cent of the total overdose burden in the area during the study period. It is impossible to declare with certainty if the SIF prevented these fatalities as it is not possible to know if overdoses occurring in the SIF would have occurred elsewhere. However, despite charges to the contrary [19], a longitudinal analysis of overdose patterns in a representative sample of SIF clients did not demonstrate that individuals took greater risks—i.e., in drug choice, mode of administration or dose—within the apparent safety of an SIF [14].

Our results are similar to those in an evaluation of the medically supervised injection centre (MSIC) in Sydney, Australia [16]. Using a similar method, they estimated that six of the 81 non-fatal overdoses in the MSIC that required naloxone during its first 18 months of operation would have resulted in a fatality, or between four and nine deaths prevented per annum, given different methodological assumptions. This rate is within the range of two to 12 per annum estimated by our methodology.

Our results differ from those in an unpublished cost-effectiveness report prepared for Canada's federal health minister [20] that estimated Insite prevented 1.08 deaths per year, an estimate below the range of the per annum calculation of our model. However, it should be noted that the report's figure was the result of an extrapolation of published and aggregated data from the first 18 months of operation; the authors were unable to benefit from SIF data from the first four years of operation; nor did their methodology account for the uncertainty surrounding several parameters. We believe the authors' population-level estimates of overdose risk differed due to the above issues as well as failure to account for the higher risk drug-using patterns of the SIF clientele [10], [21].

Obviously, the optimal strategy to evaluate the impact of the SIF on overdose rates would be to randomize participants to use (versus restricted use) of the SIF. This approach has been deemed unethical [9]. In the absence of a randomized trial, there are several approaches to assess possible effects on fatal overdose including modelling, as we have employed, and crude time series analyses comparing year-to-year changes in mortality rates. With respect to time-series approaches, previous work has identified how overdose rates are influenced by a diverse array of factors which change over time, such as drug purity. We have recently shown large fluctuations in local drug purity[22]. Other factors affecting overdose risk include age [23], drug choice [24], exposure to addiction treatment [25] and incarceration history [26]. While little work has been done on the population-level determinants of overdose mortality rates, it is reasonable to assume analogous factors are at work, including drug market dynamics, law enforcement patterns and the coverage of harm reduction measures. Because of this multiplicity of effects and the potential for unmeasured confounding due to drug supply changes in crude time series analyses, we are unaware of any method using available data that could identify the impact of the SIF on population-level overdose rates. Thus, we chose to estimate the number of averted overdose deaths using published estimates and observed, individual-level data from the SIF.

These findings have immediate policy implications. First, despite initially expressing interest in analyses of Insite's impact on local patterns of overdose [27], Canada's federal government more recently announced its opposition to the continued operation of the facility and appealed a local court ruling blocking its closure [28]. Regardless of these partisan political developments, this analysis provides evidence of the likely beneficial effect of the facility on the risk of overdose death in the DTES and echoes previous findings from Sydney's MSIC [16]. Together, these findings support the increasingly prevalent conclusion that supervised injection facilities are an effective and appropriate intervention in urban settings suffering from high levels of overdose deaths.

Our analysis has several limitations, chiefly the reliance on estimates to inform several model parameters, specifically the number of IDU in the DTES and the incidence of non-fatal overdose in the community. For the former, we relied on two previous capture-recapture studies [29], [30] and included a wide confidence interval in the Monte Carlo simulation. For the latter, we completed a sensitivity analysis to account for the wide range of non-fatal overdose rates reported in the literature. Model outputs such as the DTES fatal overdose rate and non-fatal to fatal overdose ratio are not substantially different from previous observations in other settings [31], lending credence to the estimate of averted deaths. Furthermore, in every case, we endeavoured to use conservative estimates, for example restricting the definition of a potential on-site overdose death to those characterised by a 911 call, provision of naloxone and/or an ambulance. Finally, there may be effects of the SIF that go beyond their impact on those actively using the facility. For instance, individuals can receive nurse-delivered education in safer injection practices which may reduce risk behaviours for overdose outside the facility. Over one-third of individuals report receiving this training in a representative cohort of SIF clients [32]. Although this issue requires further study, IDU who experience supervision of a nurse within the SIF may subsequently be more cautious when injecting in environments which are not supervised by trained emergency personnel [33].

In conclusion, we observed that non-fatal overdose was a common occurrence at Vancouver's SIF and, using a modelling technique based on evidence-based parameter values, we estimated that the facility prevented between eight and 51deaths over the study period. This is equal to between 37.0 per cent and 6.1 per cent of overdose fatalities in the DTES over the same time or two to 12 averted deaths per annum over the study period. These findings are consistent with analogous evaluations and support the conclusion of the facility's positive impact on public health in Vancouver's Downtown Eastside [10].

## Materials and Methods

In this analysis, we sought to estimate the number of overdose deaths averted by a supervised injection facility using methods described previously [16], [31], [34], [35]. This estimate was calculated by determining how many overdose events in the SIF would have been fatal had they happened outside the facility [16]. Specifically, the number of averted deaths is the product of the number of on-site overdoses multiplied by the ratio of fatal to non-fatal overdoses in the DTES [31], or:
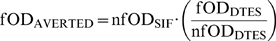
For each term in the equation, we used evidence-based estimates or direct observations. The number of overdose events in the SIF (*nfOD_SIF_*) was compiled from the facility's comprehensive on-site surveillance database. This system was the source of data for an earlier analysis of SIF overdose patterns and has already been described in detail [15]. Briefly, all new clients must register at the SIF using a pseudonymous identifier and basic information (i.e., gender and age). All activities in the SIF, including the type and amount of substances injected, the characteristics of overdose events and the interventions taken in response, are entered into the database and associated with the client's identifier.

For this analysis, we accessed a dataset from the SIF with all pseudonymous identifiers stripped. In order to limit our analysis to on-site overdose events that might conceivably resulted in a death, we restricted our definition of a potentially-fatal overdose event as any that required the provision of naloxone, a 911 call and/or an ambulance.

The ratio of fatal to non-fatal overdoses in the DTES was estimated using data from various official or peer-reviewed sources. The incidence of fatal overdose (*fOD_DTES_*) was calculated using counts of drug-induced deaths in the Downtown Eastside published annually by the British Columbia Vital Statistics Agency [36]-[44]. As these totals included deaths from drug overdose as well as suicide by drug poisoning and adverse events from medications, we multiplied the counts by 75%, the approximate proportion of deaths from drug overdoses [44]. The number of person-years at risk was calculated using estimates of the size of the IDU population in the DTES in two recent capture-recapture studies [29], [30]. The local incidence of non-fatal overdose (*nfOD_DTES_*) was estimated from a review of cross-sectional and longitudinal surveys of active IDU in both domestic and international settings [3], [5], [14], [23], [24], [31], [45]–[52]. Both rates were expressed per 1,000 person years.

A small amount of data was missing and its value was imputed. Counts of drug-induced deaths in the DTES were unavailable for 2007 and 2008. Thus, the median number of fatal overdoses per annum between 1997 and 2006 was used for both 2007 and 2008. For 2008, we multiplied this number by the proportion of the year included in our study period.

We accounted for the uncertainty in some model parameters in two ways. First, we performed a Monte Carlo simulation for each year of the study period and the entire study period. By permitting the mean, variability and distribution for each model value to be defined, Monte Carlo simulations enable the calculation of point estimates and 95% Confidence Intervals (95% CI) for model outputs. For each year in the study period we performed 10,000 iterations; for the entire study period, we performed 50,000 iterations. We have previously used the Monte Carlo method to model the impact of antiretroviral medication on mortality from HIV infection in the DTES [35] and the Americas [34]. Second, owing to the wide range of non-fatal overdose rates cited in the literature, we conducted a sensitivity analysis by repeating the Monte Carlo simulation three times, using different plausible values for the local non-fatal overdose rate: 50 per 1,000 person years, reflecting the lowest value observed in a local cohort of IDU [24]; 200 per 1,000 person years, the median value observed in a local cohort of IDU [14]; and 300 per 1,000 person years, the largest external estimate observed [31].

In a subanalyses, we calculated estimates of the number of non-fatal overdoses in the DTES for each year in the study period as well as the entire study period. These estimates are the product of the number of fatal overdoses defined in the model multiplied by the non-fatal to fatal overdose ratio. As above, we performed Monte Carlo simulations using three different non-fatal overdose rates to calculate three point estimates with 95% CI.

The evaluation of Vancouver's SIF has been reviewed and approved by the University of British Columbia/Providence Healthcare Research Ethics Board.
